# Identification of Gene Signatures for Diagnosis and Prognosis of Hepatocellular Carcinomas Patients at Early Stage

**DOI:** 10.3389/fgene.2020.00857

**Published:** 2020-07-30

**Authors:** Xiaoning Gan, Yue Luo, Guanqi Dai, Junhao Lin, Xinhui Liu, Xiangqun Zhang, Aimin Li

**Affiliations:** ^1^Integrated Hospital of Traditional Chinese Medicine, Southern Medical University, Guangzhou, China; ^2^Cancer Center, Southern Medical University, Guangzhou, China; ^3^Department of Physiology, Michigan State University, East Lansing, MI, United States

**Keywords:** hepatocellular carcinoma, transcriptome, diagnosis prediction model for early HCC, survival risk prediction model for early HCC, machine learning algorithm

## Abstract

The onset of liver cancer is insidious. Currently, there is no effective method for the early detection of hepatocellular carcinoma (HCC). Transcriptomic profiles of 826 tissue samples from the Gene Expression Omnibus (GEO), The Cancer Genome Atlas (TCGA), Genotype tissue expression (GTEx), and International Cancer Genome Consortium (ICGC) databases were utilized to establish models for early detection and surveillance of HCC. The overlapping differentially expressed genes (DEGs) were screened by elastic net and robust rank aggregation (RRA) analyses to construct the diagnostic prediction model for early HCC (DP.eHCC). Prognostic prediction genes were screened by univariate cox regression and lasso cox regression analyses to construct the survival risk prediction model for early HCC (SP.eHCC). The relationship between the variation of transcriptome profile and the oncogenic risk-score of early HCC was analyzed by combining Weighted Correlation Network Analysis (WGCNA), Gene Set Enrichment Analysis (GSEA), and genome networks (GeNets). The results showed that the AUC of DP.eHCC model for the diagnosis of early HCC was 0.956 (95% CI: 0.941–0.972; *p* < 0.001) with a sensitivity of 90.91%, a specificity of 92.97%. The SP.eHCC model performed well for predicting the overall survival risk of HCC patients (HR = 10.79; 95% CI: 6.16–18.89; *p* < 0.001). The oncogenesis of early HCC was revealed mainly involving in pathways associated with cell proliferation and tumor microenvironment. And the transcription factors including EZH2, EGR1, and SOX17 were screened in the genome networks as the promising targets used for precise treatment in patients with HCC. Our findings provide robust models for the early diagnosis and prognosis of HCC, and are crucial for the development of novel targets applied in the precision therapy of HCC.

## Introduction

Liver cancer, with the incidence (8.2% of the total cancer cases) and mortality (4.7% of the total cancer deaths) rates, is the sixth commonly diagnosed cancer and the fourth leading cause of cancer deaths among 36 cancers in the world (Bray et al., 2018). The best curative treatment plans for early hepatocellular carcinoma (HCC) patients involve surgical resection, local ablation, and liver transplantation (Llovet et al., 2016; Vibert et al., 2020), and patients who undergo such treatments usually have a relatively good prognosis, with a 5-year survival rate ranging from 60 to 80% (Bruix et al., 2016). Therefore, providing a robust and accurate tool for the diagnosis and prognosis of patients with early HCC will have a significant impact on clinical outcomes (Dhanasekaran et al., 2019).

As the amount of publicly available high-throughput data in global databases continues to grow, an open question has arisen: How can we exploit these large-scale data appropriately to achieve a comprehensive understanding of cancer at the molecular level? Machine learning (ML) is the scientific study of algorithms and statistical models and plays a critical role in various fields of human life, especially as it provides methods for diagnosis and prognosis in human diseases (Rajkomar et al., 2019; Issa et al., 2020). Several studies have applied multiple biomarkers to build prediction models for diagnosis or prognosis in clinical patients (Villanueva et al., 2011; Shi et al., 2014; Kim et al., 2019). However, the prediction accuracy and application scope of these models, which consist of predictive biomarkers, have been largely limited by sample size in previous studies.

In the present study, considering the decisive role of the sample size and tissue source in the accuracy of the model, a total of 826 patients with tumor-node-metastasis (TNM) stage I HCC from the Gene Expression Omnibus (GEO), International Cancer Genome Consortium (ICGC), Genotype tissue expression (GTEx) databases (International Cancer Genome Consortium et al., 2010; Barrett et al., 2013; Carithers and Moore, 2015), and The Cancer Genome Atlas (TCGA) were screened for the construction of models aimed at developing approaches for universal applications in early diagnosis and prognostication of HCC. Accordingly, the relationship between the variation of transcriptome profile and the oncogenic risk-score of early HCC could be investigated to clarify the potential molecular mechanism involved in the occurrence and progression of early HCC.

## Materials and Methods

### Extraction and Preprocessing of TNM Stage I HCC Transcriptome Data

**FIGURE 1:**
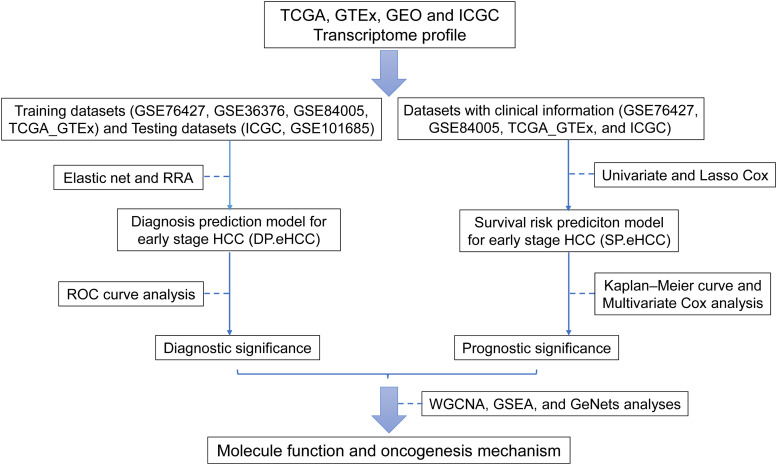
Flow diagram of the main procedure in our study. Six datasets from four international platforms were utilized to establish the diagnosis model and prognosis model. Their clinical significance and molecular mechanism were further elucidated.

The main procedure used in our research is illustrated in Figure 1. In this study, eligible datasets were searched and reviewed via the GEO^1^ database. The following strategy was used to search the GEO datasets: (Hepatocellular Carcinomas) OR (Hepatocellular Carcinoma) OR (Hepatoma) OR (Liver Cancer) OR (Adult Liver Cancer) OR (Liver Cell Carcinoma) AND “Homo sapiens.” Independent investigators (XG and AL) reviewed the eligible datasets that met the criteria and extracted the appropriate datasets. The inclusion criteria were as follows: (i) diagnosis of a stage I hepatocellular carcinoma patient based on the tumor-node-metastasis (TNM) classification system of the American Joint Committee on Cancer (AJCC); (ii) detection of expression profiling in tissue samples; and (iii) availability of original expression profiling data in both cancerous and non-cancerous specimens. The exclusion criteria were as follows: (i) datasets from research on cell lines or animals; (ii) cancerous or non-cancerous groups with small sample sizes (*n* < 5); and (iii) expression datasets without gene expression data, such as non-coding RNA profiling by array, methylation profiling by array, and so on. Discrepancies between the decisions of the two investigators were resolved by discussions among all authors.

Moreover, processed GEO data were fetched using the R package GEOquery (Davis and Meltzer, 2007), and microarray probes were transformed to Entrez Gene IDs using the R package biomaRt (Durinck et al., 2009). For these microarray probes, if multiple probes are mapped to the same Entrez Gene ID, the expression value for the Entrez Gene ID is calculated as the median of the expression values of those probes. RNA-Seq datasets (“TCGA_liver” and “gtex_liver”) of TCGA and GTEx^2^ were extracted from R package recount^3^ (Collado-Torres et al., 2017). The batch effects between TCGA and GTEx normalized data were analyzed by tSNE analysis and corrected by ComBat of the R package sva (Johnson et al., 2007). The merged TCGA_GTEx dataset was used for the down-stream analyses. The JP Project from International Cancer Genome Consortium (ICGC-LIRI-JP) collected the RNA-Seq data and clinical information of HCC patients, and this ICGC dataset was extracted from Database of Hepatocellular Carcinoma Expression Atlas (HCCDB) (Lian et al., 2018).

### Construction of the DP.eHCC Model

Elastic net, a generalization of ridge regression and the Lasso, is a regularization method that is used to fit a generalized linear model via the function of the R package glmnet (Friedman et al., 2010). The eligible datasets (TCGA_GTEx, GSE76427, GSE36376, GSE84005, GSE101685, and ICGC) were analyzed by using the elastic net. These datasets were split into two groups: training datasets (GSE76427, GSE36376, GSE84005, and TCGA_GTEx) and testing datasets (GSE101685 and ICGC). Next, training datasets were merged after the batch effects from each dataset were adjusted by ComBat (Johnson et al., 2007). In order to use the elastic net, the expression data were reduced to the set of genes that were common to all datasets being merged, because it was possible for each dataset to have expression values for a slightly different set of genes. The penalty (α = 0.9) of the elastic net was utilized to fit a generalized linear model. And, the elastic net is used to perform cross-validation. One of the results of cross-validation is a value for the regularization parameter lambda, which determines how much shrinkage is used to train the model. In addition, leave-one-study-out cross-validation was used for the classifier test in each training group dataset, and this classifier was then tested for each testing group dataset (Hughey and Butte, 2015).

Differential gene expression analysis between hepatocellular carcinoma tissues and non-cancerous liver tissues was performed using the R package limma (Ritchie et al., 2015) for training datasets (GSE76427, GSE36376, GSE84005, and TCGA_GTEx). The overlapping differentially expressed genes (DEGs) from these datasets were identified by robust rank aggregation (RRA) method of the R package RobustRankAggreg (Kolde et al., 2012). DEGs were distinguished by having log_2_ fold change > 1 and adjusted *p*-value < 0.05. As the predictors for early HCC diagnosis with the most confidence, the DEGs intersecting between the RRA method and the elastic net penalty method were picked up by the R package Venn Diagram. The combination of these predictors was analyzed by logistic regressions to generate the formula for the construction of the diagnosis prediction model for early HCC (DP.eHCC).

### Construction of the SP.eHCC Model

Eligible datasets (GSE76427, TCGA_GTEx, and ICGC) with survival information were used for the survival analysis. A univariate Cox analysis was performed to assess the prognostication genes for predicting the overall survival (OS) of early HCC patients. Prognostication genes with a Univariate Cox value of *p* < 0.05 were further screened with the least absolute shrinkage and selection operator (Lasso) Cox model (Tibshirani, 1997) by utilizing the R package glmnet. Moreover, the genes screened by the Lasso Cox regression analysis with min lambda were utilized to construct a survival risk prediction model for early HCC (SP.eHCC). The formula of SP.eHCC was established by calculating the expression levels of selected genes weighted by their corresponding coefficients. The relationship between the risk score of overall survival and the prognostic genes of the SP.eHCC model was illustrated by risk score distribution, scatter plot, and gene expression heatmap.

### WGCNA for the Transcriptome Data of Early HCC

The Weighted Correlation Network Analysis (WGCNA) (Langfelder and Horvath, 2008) was utilized to build the weighted gene co-expression correlation network, and the distances between different transcripts were calculated using the “Pearson” correlation coefficient. Construction of the WGCNA network and detection of the co-expressed gene modules were conducted using an unsigned topological overlap matrix (TOM), a β power of 3, and a minimal module size of 30. By evaluating the relationships among co-expression gene modules and clinical parameters including gender, age, DP.eHCC, and SP.eHCC, we were able to identify the modules that were highly correlated with the clinical parameters of HCC patients. The modules (with the highest correlation coefficient among all the modules) correlated with DP.eHCC and SP.eHCC (positively or negatively) were selected for the further analyses.

### GSEA and GeNets Analyses

Gene Set Enrichment Analysis (GSEA)^4^, was used to identify the significant KEGG pathways enriched in the modules highly correlated with DP.eHCC and SP.eHCC. Co-expressed genes in the modules selected by WGCNA analysis were ranked by the Pearson correlation coefficient between gene expression and the fraction of clinical traits. The statistically significant (*p* < 0.05) pathways enriched in the gene set of modules correlated with the DP.eHCC and SP.eHCC were visualized using the R package clusterProfiler (Yu et al., 2012). Additionally, the co-expressed genes in WGCNA modules were used to construct the genome networks (GeNets) mapped by the pathways of GSEA. And, this molecular regulatory network was utilized to illustrate the potential oncogenes and corresponding pathways involved in the modules correlated with DP.eHCC and SP.eHCC using the GeNets platform (Li et al., 2018).

### Statistical Analyses

Statistical analyses of this study were conducted using R software (version 3.5.2)^5^ and SPSS software (version 22.0). Receiver operating characteristic (ROC) curve analysis with area under the curve (AUC) was utilized to assess the predictive performance of DP.eHCC and its DEG members via the R package pROC. In order to appraise the prognostic performance of early HCC patients with different clinical parameters including gender, age (cut-off value by 50), SP.eHCC (utilizing the median risk score as the cutoff value), Kaplan–Meier curves with the log-rank test were performed using the R package survival. Additionally, univariate Cox regression and multivariable Cox regression analyses were utilized to confirm the independent prognostic factors within clinical pathological characteristics including gender, age, and SP.eHCC. Furthermore, based on the identified prognostic factors confirmed by multivariate Cox analysis, a nomogram was utilized to predict the 1-, 3-, and 5-year overall survival probabilities in early HCC. Calibration of the nomogram was evaluated graphically by calibration curves and determined by the concordance index (C-index).

## Results

In order to explore the diagnostic and prognostic prediction methods for early hepatocellular carcinoma (HCC), a total of 826 cases of cancerous or non-cancerous liver tissue specimens with early HCC from six merged datasets (GSE76427, GSE36376, GSE84005, GSE101685, TCGA_GTEx, and ICGC) were included in our study (Figure 1 and Supplementary Table S1). Additionally, the batch effect between TCGA and GTEx was visualized and adjusted by tSNE and ComBat, respectively (Supplementary Figure S1). And, the batch effects among those six eligible datasets were visualized using tSNE (Supplementary Figure S2), further analyses were conducted after the batch effects among each eligible dataset were adjusted by ComBat.

### Diagnostic Prediction Performance of the DP.eHCC Model in Early HCC

**FIGURE 2:**
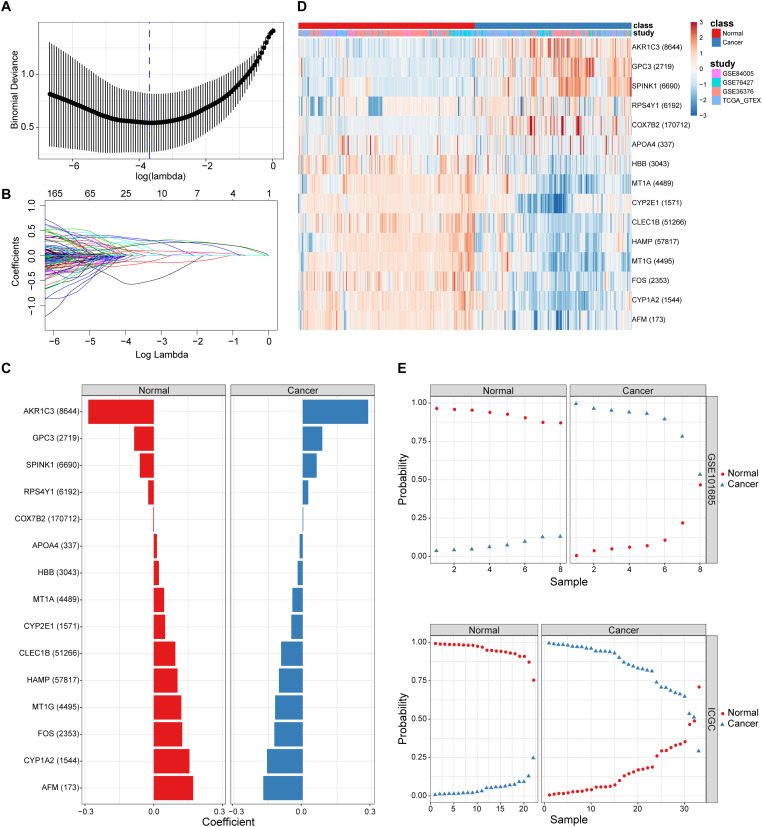
The screening and validation of 15 genes conducted by the diagnostic classifier. **(A,B)** Binomial deviance as a function of the regularization parameter lambda for leave-one-study-out cross-validation on the training datasets. Points correspond to the means, and error bars correspond to the standard deviations. Coefficients of 15 genes were selected by the lambda with the minimum binomial deviance marked by the blue dashed line (lambda = 0.025, ln(lambda) = −3.692). **(C)** Coefficient values for each of the fifteen selected genes. A positive coefficient for a gene signature within its class indicates that elevated expression of this gene increases the probability of a specimen belonging to its tissue type. **(D)** Heatmap for describing the expression levels of selected genes in the binomial classifier erected by training datasets. Each row is a gene with its Entrez Gene ID in parentheses; each column is a sample. **(E)** Estimated probabilities for samples in testing datasets (GSE101685 and ICGC). For each sample, there are two points, corresponding to the probability that the sample belongs to the respective class. Within each dataset and class, samples are sorted by the probability of the true class. For most samples, the probability of the true subtype is near 1, indicating an unambiguous classification.

By combining the elastic net and robust rank aggregation (RRA) analysis, the DP.eHCC model was constructed to provide early diagnosis method for early HCC. As shown in Figure 2, using the value of the regularization parameter that gave the lowest binomial deviance, we identified a binomial classifier on all samples from the training datasets (Figure 2A). This classifier was erected based on the expressive signatures of 15 genes (Figure 2B), and genes with non-zero coefficients for each class were found to be almost mutually exclusive (Figure 2C and Supplementary Table S2). The heatmap showed the differential expression levels of the 15 genes in cancerous and non-cancerous liver tissues across multiple training datasets (Figure 2D). The overall accuracy (fraction of correctly classified samples) of the binomial classifier for cross-validation on training datasets was 90.6% (Supplementary Figure S3 and Supplementary Table S3). To further validate our method, we also evaluated the classifier on two independent testing datasets (Figure 2E). Across the two testing datasets, the overall accuracy was 98.6% (Supplementary Table S4). These results indicated that our method can successfully extract a robust signal from gene expression data derived from multiple platforms.

**FIGURE 3:**
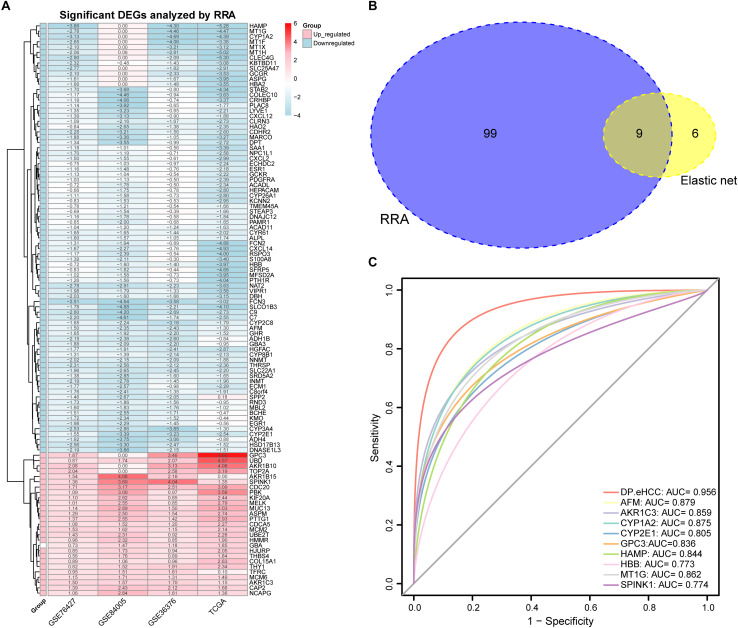
Diagnostic performance of the DP.eHCC model selected by elastic net and RRA. **(A)** Heatmap showing the top 27 up-regulated genes and top 81 down-regulated genes in the training datasets (logFC > 1, adjusted *p* < 0.05). Each row represents one gene and each column indicates one dataset. Red indicates up-regulation and light blue represents down-regulation. DEGs: differentially expressed genes; RRA: robust rank aggregation. **(B)** As illustrated in the Venn Diagram, nine robust DEGs (AFM, AKR1C3, CYP1A2, CYP2E1, GPC3, HAMP, HBB, MT1G, and SPINK1) were identified by the intersection genes from the RRA (blue) and elastic net (yellow) analyses. **(C)** Receiver operating characteristic (ROC) curve analyses of the DP.eHCC model and its gene members for early HCC diagnosis. When compared with each gene member of the DP.eHCC model, the prediction efficiency of the DP.eHCC model was shown to be significantly enhanced (AUC = 0.956, *p* < 0.001).

A total of 27 up-regulated and 81 down-regulated significantly differentially expressed genes (DEGs) were identified by RRA analysis, and these genes were split into red and light blue groups, respectively (Figure 3A). Next, from the results of the elastic net and RRA analysis, nine DEGs were selected by a Venn diagram for building the DP.eHCC model (Figure 3B). The risk score formula consisting of nine DEGs was established as follows: DP.eHCC (risk score) = 1.7986040–0.4530214 × expression level of AFM + 0.7464234 × expression level of AKR1C3–0.1653185 × expression level of CYP1A2–0.2039676 × expression level of CYP2E1 + 0.5878815 × expression level of GPC3–0.2612360 × expression level of HAMP–0.3634324 × expression level of HBB–0.1410460 × expression level of MT1G + 0.1215430 × expression level of SPINK1.

Furthermore, the predictive performance of the DP.eHCC model and its gene members in 826 total cases of early HCC was verified by ROC curves. The AUC of DP.eHCC model for the diagnosis of early HCC was 0.956 (95% CI: 0.941–0.972; *p* < 0.001) with a sensitivity of 90.91%, a specificity of 92.97%, and a diagnostic threshold value of 0.0324 (Figure 3C). The results showed that the DP.eHCC model significantly improved the prediction performance over its nine differentially expressed genes alone, including the following AUC values: AFM—0.8787 (95% CI: 0.8538–0.9037; *p* < 0.001); AKR1C3—0.8588 (95% CI: 0.8319–0.8856; *p* < 0.001); CYP1A2—0.8753 (95% CI: 0.8495–0.901; *p* < 0.001); CYP2E1—0.8045 (95% CI: 0.7723–0.8368; *p* < 0.001); GPC3—0.8358 (95% CI: 0.8057–0.8658; *p* < 0.001); HAMP—0.8440 (95% CI: 0.8156–0.8724; *p* < 0.001); HBB—0.7728 (95% CI: 0.7406–0.8050; *p* < 0.001); MT1G—0.8617 (95% CI: 0.8333–0.8901; *p* < 0.001); and SPINK1—0.7740 (95% CI: 0.7409–0.8071; *p* < 0.001) (Figure 3C). Additionally, the diagnosis performance of the DP.eHCC model in HCC patients was also validated in TCGA and ICGC cohort, the results showed the DP.eHCC model also achieved a well diagnosis performance in HCC from the independent database (Supplementary Figure S4).

### Prognostic Prediction Performance of the SP.eHCC Model in Early HCC

**FIGURE 4:**
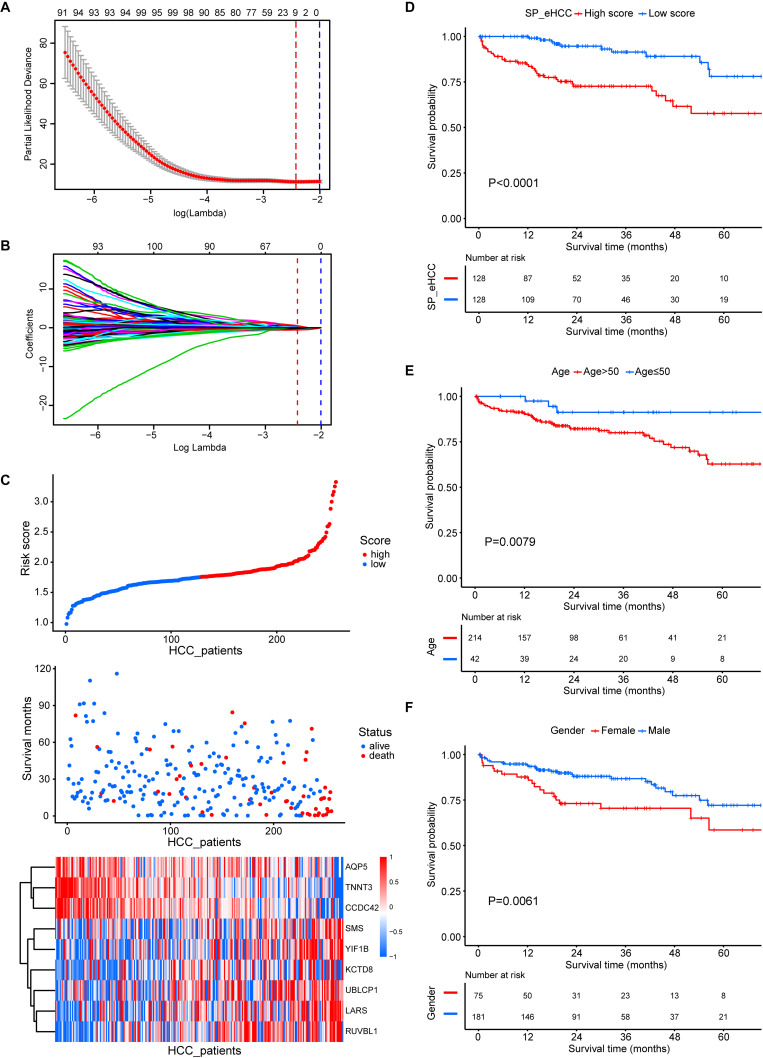
Prognostic significance of the SP.eHCC model and other clinical parameters in early stage hepatocellular carcinoma (HCC). **(A,B)** Lasso Cox analysis identified nine genes at lambda with minimum partial likelihood deviance (red dotted line) that correlated with the overall survival of early stage HCC patients in the merged cohort (GSE76427, TCGA_GTEx, and ICGC). The red vertical dashed lines indicate the lambda min. **(C)** The relationship between the risk score of overall survival and the expression of nine genes (AQP5, TNNT3, CCDC42, SMS, YIF1B, KCTD8, UBLCP1, LARS, and RUVBL1) in the SP.eHCC model was shown in the risk score distribution (top), scatter plot of survival status (middle), and heatmap of the prognostic 9-gene signature (bottom) in patients with HCC. The pseudocolors on the right of the heatmap plot represent expression levels from low to high on a scale from –1 to 1, ranging from a low correlation power (white) to high (blue, or red). **(D–F)** Kaplan–Meier curves of overall survival for 256 early stage HCC patients with different clinical parameters including SP.eHCC, Age, and Gender. HCC patients with relatively low-risk scores had longer mean survival times than patients with relatively high-risk scores (*p* < 0.0001). Patients aged ≤ 50 had longer mean survival times than patients aged > 50 (*p* = 0.0079). Male patients had longer mean survival times than female patients (*p* = 0.0061).

All of the 256 early HCC patients collected from the merged cohort (GSE76427, TCGA_GTEx, and ICGC) were included in the overall-survival analysis. Among 1344 prognosis-related genes (Supplementary Table S5, *p*-value < 0.05) screened by univariate Cox regression, nine genes were further selected for SP.eHCC model construction using the minimizing λ method of the Lasso Cox analysis (Figures 4A,B). The prognostic risk score formula consisting of these nine genes was established as follows: SP.eHCC (risk score) = 0.2609 × expression level of UBLCP1–0.4423 × expression level of CCDC42–0.1963 × expression level of AQP5 + 0.0717 × expression level of KCTD8 + 0.3821 × expression level of LARS + 0.2059 × expression level of SMS–0.5612 × expression level of TNNT3 + 0.4541 × expression level of RUVBL1 + 0.5156 × expression level of YIF1B.

The correlations between the risk scores of 256 ordered patients and 9-gene expression patterns are illustrated. These results suggest that as the risk score of patients increased, the number of death events accumulated, and risk markers (coefficient of gene > 0) exhibited increased expression, while protective markers (coefficient of gene < 0) exhibited decreased expression (Figure 4C). Kaplan–Meier curves were used to evaluate the relationships among clinical parameters (SP.eHCC, age, and gender) and the overall survival of patients. When a median value of 1.755 was selected as the SP.eHCC risk score level threshold, early HCC patients with relatively low risk scores (*n* = 128) had longer mean survival times than patients with relatively high risk scores (*n* = 128) (95.705 ± 5.642 months vs. 55.901 ± 3.763 months, *p* < 0.0001) (Figure 4D). Patients aged ≤ 50 (*n* = 42) had longer mean survival times than patients aged > 50 (*n* = 214) (102.078 ± 4.535 months vs. 70.472 ± 5.665 months, *p* = 0.0079) (Figure 4E). Male patients (*n* = 181) had longer mean survival times than female patients (*n* = 75) (85.504 ± 4.641 months vs. 65.516 ± 7.326 months, *p* = 0.0061) (Figure 4F). The results of multivariable Cox regression analysis showed that SP.eHCC model performed best for predicting the overall survival risk of HCC patients (HR = 10.79; 95% CI: 6.16–18.89; *p* < 0.001) compared with gender (HR = 0.47; 95% CI: 0.27–0.85; *p* = 0.012), and age (HR = 1.01; 95% CI: 0.99–1.04; *p* = 0.272). Notably, the results indicated that age could not be considered as a prognostic predictor for early HCC patients (Table 1).

**TABLE 1:** Cox analysis of clinicopathological parameters for overall survival in HCC.

**Variables**	**Univariate**			**Multivariate**		
	**HR**	**95% CI**	***p* value**	**HR**	**95% CI**	***p* value**
Age	1.04	1.01–1.06	0.005*	1.01	0.99–1.04	0.272
Gender	0.46	0.26–0.81	0.007*	0.47	0.27–0.85	0.012*
SP.eHCC	11.65	6.81–19.91	*p < 0.001	10.79	6.16–18.89	*p < 0.001

HR, hazard ratio; 95% CI, 95% confidence interval; HCC, hepatocellular carcinoma; SP.eHCC, survival risk prediction model for early HCC. *Statistically significant (*p* < 0.05).

**FIGURE 5:**
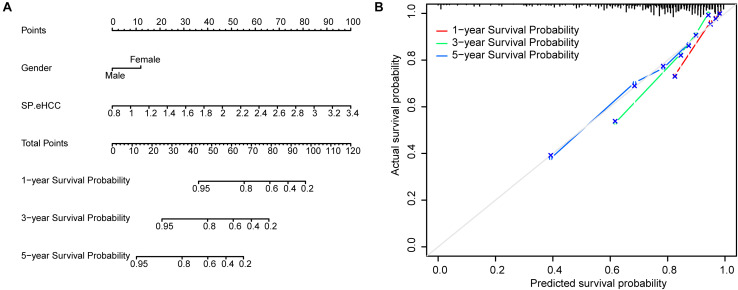
Prognostic significance of overall survival predictors validated in nomogram model. **(A)** A nomogram was established to predict the risk score and survival probability of early HCC patients. Survival risk factors including SP.eHCC and gender were integrated in the nomogram. **(B)** The comparison between predicted and actual outcomes for 1-, 3-, and 5-year survival probabilities in the nomogram is shown in the calibration plots.

To further test the coefficient prediction efficiency of overall survival predictors validated by multivariable Cox regression analysis, including gender and SP.eHCC. A nomogram model was established in 256 early HCC patients. The results showed that the overall score of the nomogram was helpful for providing a quantitative method to accurately predict the prognosis of early HCC patients (1-, 3-, and 5-year survival probabilities) (Figure 5A). The prediction probability and actual probability of 1-, 3-, and 5-year survival in the calibration curve showed satisfactory overlap, indicating a good agreement (Figure 5B), and the C-index of this nomogram was 0.841 (95% CI: 0.789–0.893; *p* < 0.001).

### Molecular Mechanism Underlying the Oncogenesis of Early HCC

**FIGURE 6:**
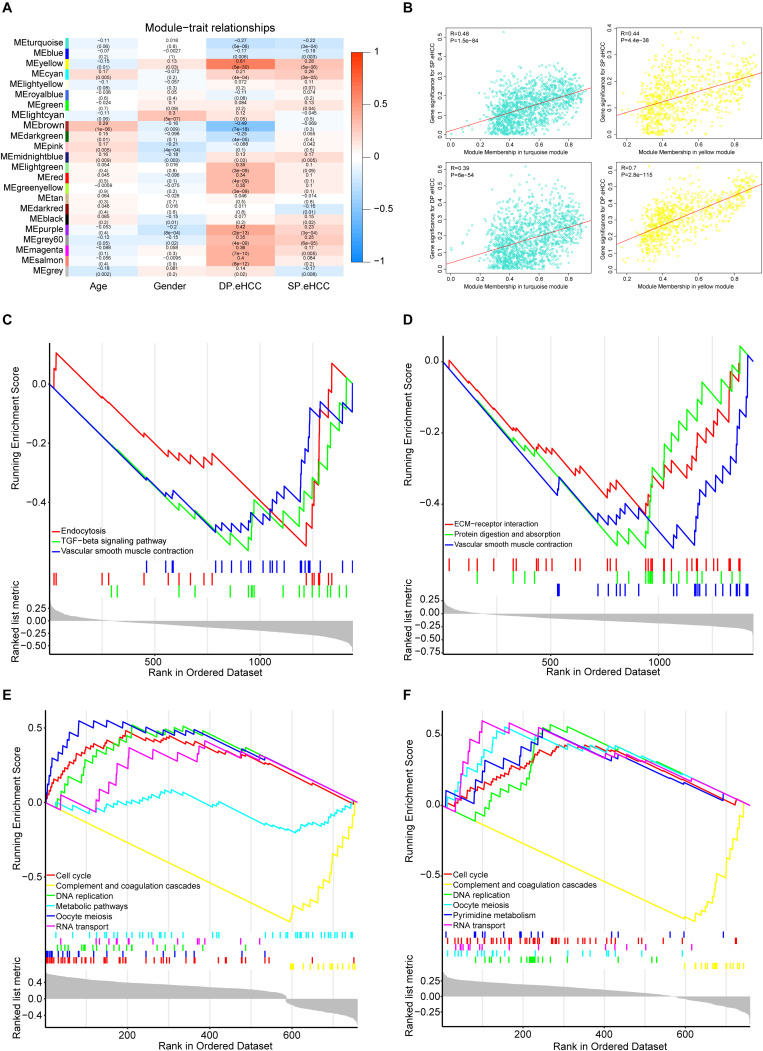
Identification of the functional pathways of modules related to the DP.eHCC, and SP.eHCC. **(A)** Weighted Correlation Network Analysis (WGCNA) showing correlations between module eigengenes and clinical traits of early-stage HCC. Each block contains the correlation coefficient and *p* value. **(B)** Verification of the WGCNA gene modules highly correlated with clinical traits. The scatter plots were utilized to illustrate the correlations of gene significance for clinic traits versus module members in the two modules including the turquoise and yellow modules. The “Pearson” score and *p* value of each module are shown in the top left of each figure. **(C,D)** Enrichment plots showing the KEGG gene sets (*p* < 0.05) enriched by co-expressed genes of the turquoise module correlated with DP.eHCC and SP.eHCC, respectively. **(E,F)** Enrichment plots showing the KEGG gene sets (*p* < 0.05) enriched by co-expressed genes of the yellow module correlated with DP.eHCC and SP.eHCC, respectively. Enrichment score (ES): A positive ES indicates gene set enrichment at the top of the ranked list; a negative ES indicates gene set enrichment at the bottom of the ranked list. The ranking metric measures a gene’s correlation with a phenotype.

To investigate the mechanism of oncogenesis and progression of early HCC, we performed WGCNA on the merged expression matrix (GSE76427, GSE84005, TCGA_GTEx and ICGC) in 275 early HCC patients with clinical traits including age, gender, DP.eHCC, and SP.eHCC. The expression levels of 11,853 genes in this matrix were implemented to build a co-expression network. By setting the soft-thresholding power as 3 (scale free *R*^2^ = 0.83), we eventually identified 22 modules (Supplementary Figure S5; non-clustering genes shown in gray). The relationships between the clinical traits and the eigenvalue of each module are presented in the heatmap (Figure 6A). From the heatmap of module-trait correlations, we identified two modules, including a turquoise module (1452 genes), and yellow module (776 genes), which were significantly highly correlated with clinical traits, including SP.eHCC and DP.eHCC (Figure 6B).

For a better understanding of the molecular functions underlying the oncogenesis of early HCC, Gene Set Enrichment Analysis (GSEA) was applied to analyze the possible functional pathways of co-expressed genes in the two modules (turquoise and yellow) highly correlated with DP.eHCC and SP.eHCC. For co-expressed genes in the turquoise module, the significant pathways (*p* < 0.05) including “TGF-beta signaling pathway,” “Endocytosis,” and “Vascular smooth muscle contraction,” were negatively correlated with DP.eHCC. And, the significant pathways (*p* < 0.05) including “Vascular smooth muscle contraction,” “Protein digestion and absorption,” and “ECM-receptor interaction,” were negatively correlated with SP.eHCC (Figures 6C,D). For co-expressed genes in the yellow module, we discovered four significant pathways (*p* < 0.05) including “cell cycle,” “DNA replication,” “oocyte meiosis,” and “RNA transport” that were positively correlated with DP.eHCC and SP.eHCC simultaneously. And, both DP.eHCC and SP.eHCC were negatively correlated with “complement and coagulation cascades” (*p* < 0.05). Additionally, DP.eHCC was negatively correlated with “Metabolic pathways” (*p* < 0.05), and SP.eHCC was positively correlated with “Pyrimidine metabolism” (*p* < 0.05) (Figures 6E,F).

**FIGURE 7:**
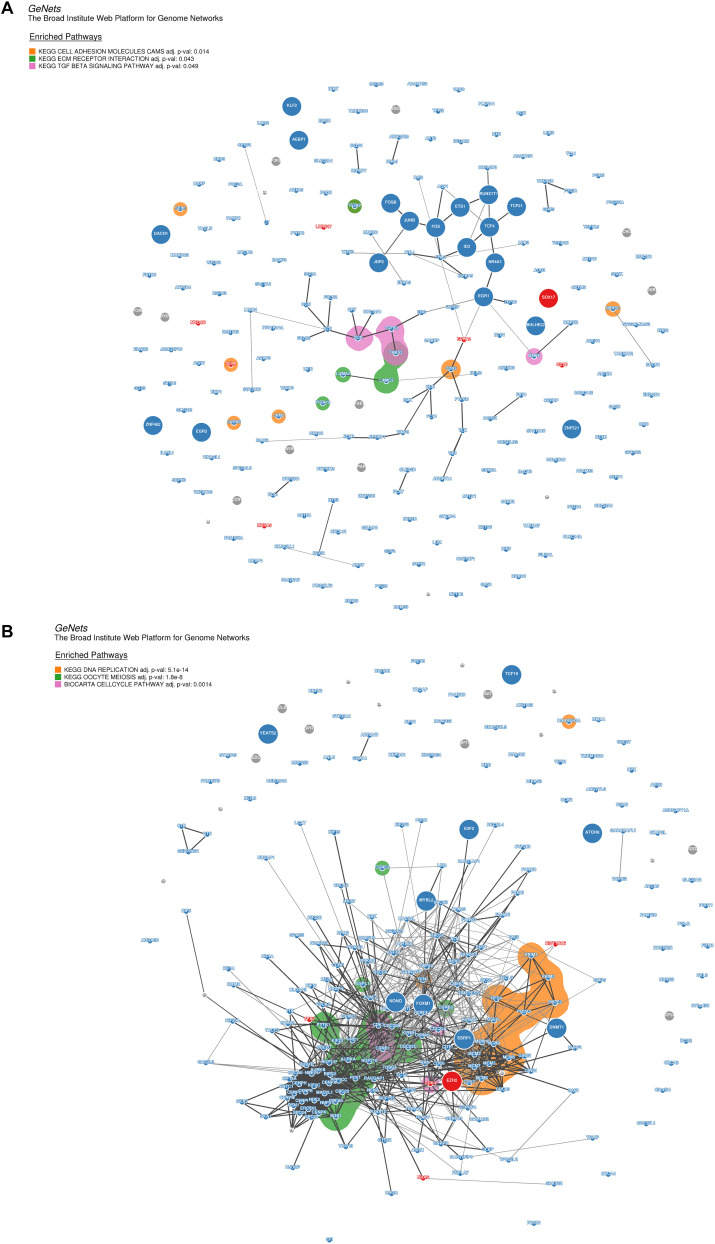
Genome networks analysis of co-expressed genes in modules of weighted correlation network analysis **(A)** The genome network of co-expressed genes in turquoise module **(B)** The genome network of co-expressed genes in yellow module. Network nodes represent proteins (Size: large nodes represent transcription factor and small nodes do not represent transcription factor; Color: red module means having significant protein-altering mutations, blue module means having no significant protein-altering mutations, gray means not assigned), and network edges represent protein-protein associations (The edges have a score between (0,1) and the encoding is gray scale. The edges with higher scores represent darker edges and the edges with smaller scores represent lighter edges). KEGG pathways of nodes are based on the 853 expertly curated pathways from the Molecular Signatures Database (MSigDB), respectively. Overlay the enriched KEGG pathways on the network using bubble sets and code the square with different color.

Moreover, for the purpose of identifying the hub genes that play crucial roles in the molecular regulation network involved in the pathways of the modules highly correlated to DP.eHCC and SP.eHCC, we selected the co-expressed genes from two modules (turquoise and yellow) to construct the genome networks (GeNets). For the turquoise module, we built a genome network which was mapped by three pathways: “Cell adhesion molecules (CAMs),” “ECM-receptor interaction,” and “TGF-beta signaling pathway.” EGR1 (the transcription factor without significant protein-altering mutations) and SOX17 (the transcription factor with significant protein-altering mutations) were selected as the molecules regulating the oncogenesis of HCC (Figure 7A). For the yellow module, we built a genome network which was mapped by three cell proliferation pathways (KEGG): “cell cycle,” “DNA replication,” and “oocyte meiosis.” EZH2 (the transcription factor with significant protein-altering mutations) was selected in the molecular regulation network most probably regulating the oncogenesis of HCC (Figure 7B). These results indicate that the oncogenesis of early HCC is mainly mediated by pathways associated with cell proliferation and tumor microenvironment.

## Discussion

Delayed diagnosis is a major factor responsible for the poor prognosis of hepatocellular carcinoma (HCC). Therefore, developing a novel strategy for early detection of HCC could improve outcomes of patients with HCC (Marrero et al., 2018; Ayoub et al., 2019). Alpha-fetoprotein (AFP) performs disappointingly in early HCC screening and surveillance because of its low sensitivity and specificity (Marrero et al., 2009; El-Bahrawy, 2010). Compared with AFP, GPC3 performs better in the early detection of HCC, and its capacity for diagnosis is not affected by the tumor size and stage (Tangkijvanich et al., 2010). More importantly, *GPC3* can even distinguish dysplastic nodules in cirrhosis from early HCC (Llovet et al., 2006). However, the predictive performance of individual biomarkers is impaired by the high heterogeneity of HCC. Consequently, a combination of multiple biomarkers and further clinical tests is recommended to boost the early diagnosis of HCC (Chaiteerakij et al., 2015).

There are several methods to build the multiple linear regression model, as each method is suitable for a given dataset with specific features. However, the bias of multiple linear regression model is dependent on the response variable (*n*) and the predictive variable (*p*). The character of our data has a statistical frame of 826 early HCC samples and more than 10,000 independent variables. In view of previous studies (Engebretsen and Bohlin, 2019; Du et al., 2020), elastic net is known to work better for this data type of our study that has much more independent variables than dependent variables (*n* < < *p*). By using the elastic net and RRA analysis, we screened nine gene expression signatures, including *AFM*, *AKR1C3*, *CYP1A2*, *CYP2E1*, *GPC3*, *HAMP*, *HBB*, *MT1G*, and *SPINK1*, to construct a diagnosis prediction model for early HCC (DP.eHCC). Compared with *GPC3* or other independent gene signatures, the diagnosis efficiency of DP.eHCC in our study greatly improved in 826 cases of early HCC patients (AUC = 0.956; 95% CI: 0.941–0.972; *p* < 0.001). In addition to *GPC3*, most gene signatures used in our diagnosis model have also been confirmed in liver cancer (Wu et al., 2000; Chen et al., 2014; Ji et al., 2014; Li et al., 2015; Zhao et al., 2019), and the expression trends of those genes are consistent with our DP.eHCC model. In order to provide a robust indicator for the prognostic evaluation of early HCC, we also constructed a prognostic model, named SP.eHCC (HR = 10.79; 95% CI: 6.16–18.89; *p* < 0.001). This prognostic model consists of nine genes, including *UBLCP1*, *CCDC42*, *AQP5*, *KCTD8*, *LARS*, *SMS*, *TNNT3*, *RUVBL1*, and *YIF1B*. We clearly illustrated the impacts of these nine gene expression levels on the overall survival risk of HCC patients by a combination of the risk score distribution, survival status scatter plot, and gene expression heatmap (see Figure 4C). Furthermore, our study indicated that male patients with early HCC could achieve longer overall survival times than female patients with early HCC. In consideration of the synergistic role of clinical parameters (age, gender, and SP.eHCC) in the overall survival condition of early HCC patients, we further put these clinical parameters into the Cox and nomogram model analysis. The nomogram model was certified to perform well for predicting the 1-, 3-, and 5-year survival rates of patients, showing a C-index of 0.841 (95% CI: 0.789–0.893; *p* < 0.001).

In recent years, some researchers have applied machine learning algorithms to provide methods for the early detection of HCC. Shi et al. identified a three-gene model with an AUC of 0.96 (95% CI: 0.93–0.99) for early HCC diagnosis based on six differentially expressed genes (DEGs) sifted by a microarray analysis of peripheral blood mononuclear cell (PBMC) samples from 26 patients (Shi et al., 2014). Kim et al. identified a five-metabolite (methionine, proline, ornithine, pimelylcarnitine, and octanoylcarnitine) model for early HCC diagnosis with serum samples, and their model distinguished 53 HCC patients from 47 cirrhosis patients and 50 normal controls, with an area under the receiver operating curve (AUC) of 0.82 in the training group. They tested the five-metabolite model in 82 HCC and 80 cirrhosis patients, and the performance of their model was also demonstrated to have a good performance with an AUC of 0.94 in the testing group (Kim et al., 2019). However, the accuracy of the statistical data and the diagnosis performance stability of their models were limited by the study population, sample size, and tissue type in their research.

Up to date, (Cai et al., 2019) developed a 5 hmc diagnostic model by using the elastic analysis of genome data of early HCC, and their research showed promising to boost the current knowledge of diagnosis of early HCC. Compared with the limitation of their study, our study has its own novelty and advantages. Firstly, our study conducted elastic net analysis of a large early HCC cohort with various population from international multi-platforms, which makes our predictive models more compatible for universal applications in early diagnosis and prognostication of HCC. Secondly, our diagnosis prediction model was established based on the differentially expressed genes generated in the cancerous and non-cancerous liver tissue samples of early HCC patients, which could be a more credible way for explaining the alterations in hepatocellular carcinogenesis. Consequently, the oncogenic risk-score of early HCC could be utilized to investigate the potential molecular mechanism involved in the pathogenesis of early HCC. Kaur et al. (2019) performed a universal multiple-platform transcriptome analysis, and identified three genes (FCN3, CLEC1B, and PRC1) for diagnosis and prognosis of HCC. Liu et al. (2019) developed six gene signatures and nomogram model to predict overall survival of HCC by using the lasso Cox analysis of HCC cohort from global databases, and the predictive model established in their study showed a good performance in prognosis of HCC. However, at the perspective of clinical application, they might neglect a critical factor that the genomic variation of HCC patients will be largely affected by various treatment measures for late-stage HCC patients including radiotherapy, chemotherapy, or combination. Thus, they need to take this into consideration when they established their genomic prognosis model. Compared with their study, we selected the TNM stage I HCC cohort for the establishment of prognosis model so that our model could be erected with the minimum influence by those factors, including the intervention measures and tumor genetic alteration of HCC.

The oncogenesis mechanism involved in early HCC is determined by the complex interactions of biological molecules. Comprehensive analysis of the molecular regulatory network via exploring the variation of transcriptome profile will help explain the hepatocarcinogenesis process. By utilizing Weighted Correlation Network Analysis (WGCNA), Gene Set Enrichment Analysis (GSEA), and genome networks (GeNets) analyses, we explored the molecular mechanisms responsible for elucidating the pathogenesis of HCC to provide crucial evidence for the molecular targeted therapy of early HCC. On one hand, both DP.eHCC and SP.eHCC are negatively correlated with the co-expressed genes in yellow module, which are significantly enriched in pathways closely associated with cell proliferation (“cell cycle,” “DNA replication,” and “oocyte meiosis”). And the GeNets analysis indicates that those cell proliferation pathways are most probably regulated by enhancer of zeste homologue 2 (*EZH2*). *EZH2*, as a master regulator of transcription, plays a critical role in occurrence and progression of human cancers (Kim and Roberts, 2016). *EZH2* has been unraveled as a core factor in hepatocarcinogenesis, self-renewal of liver cancer stem cells (CSCs), and molecular targeted therapy (Cheng et al., 2011; Zhu et al., 2016; Xiao et al., 2019). However, the regulatory mechanism of *EZH2* in oncogenic transformation remains unclear. Our study hence provides the evidence for elucidating the oncogenesis of HCC based on the regulatory network of *EZH2*.

On the other hand, both DP.eHCC and SP.eHCC are negatively correlated with the co-expressed genes in turquoise module, which are significantly enriched in pathways, including “Cell adhesion molecules (CAMs),” “ECM-receptor interaction,” and “TGF-beta signaling pathway.” Those pathways were closely associated with immune and tumor microenvironment (TME) of liver (Harjunpaa et al., 2019; Hintermann and Christen, 2019). Moreover, the GeNets analysis indicates that those pathways are most probably mediated by early growth response 1 (EGR1). The protein encoded by *EGR1* is a nuclear protein and functions as a transcriptional regulator. EGR1 was confirmed as a cancer suppressor by targeting *CD24A* in HCC (Li et al., 2019). Compared with the *EGR1*, SRY-box transcription factor 17 (*SOX17*) has been confirmed as the transcription factor with significant protein-altering mutations by WGCNA and GeNets. *SOX17* could encode a member of the SOX (SRY-related HMG-box) family of transcription factors involved in the regulation of embryonic development and in the determination of the cell fate, and inhibit human HCC cells growth via negatively regulating the β-catenin/Tcf-dependent transcription (Jia et al., 2010). Expression of *SOX17* could induce tuft cells express the tumorigenic factors that can alter the TME in mice (Delgiorno et al., 2014), but the relationship of *SOX17* with TME-related pathways is still not clear in the oncogenesis of HCC. Thus, our research provides evidence to identify the potential relationship of *SOX17* with the TME-related pathways in the oncogenesis of early HCC. Nevertheless, we established the transcriptional regulatory network of molecules annotated by functional pathways for illustrating the occurrence and progression of early HCC. Further researches are still required to verify the role of *EZH2*, *EGR1*, and *SOX17* for the molecular targeted therapies of early HCC patients through *in vitro* and *in vivo* experiments.

Our predictive models will be promoted through overcoming the following limitations: Firstly, batch effects are still the important factor for the comprehensive analysis of large cohort of early HCC from multi-platforms, although we reduced the influence of batch effects in our research using Combat (Johnson et al., 2007). Secondly, the TNM staging of AJCC system fails to account for the degree of liver dysfunction and patient’s poor performance status (Marrero et al., 2018), which results in those clinical factors not to be considered in our research. Therefore, it is of significance to verify the performance of our diagnosis and prognosis models in patients with early HCC defined by the other staging systems.

## Conclusion

We established the robust prediction models (DP.eHCC and SP.eHCC) for the diagnosis and prognosis of early hepatocellular carcinoma (HCC). Moreover, based on molecular regulatory relationships and functional pathway annotations of the transcriptome profile, we comprehensively analyzed the molecular mechanism involved in occurrence and progression of early HCC. It was clarified that the oncogenesis and poor prognosis of early HCC are mainly caused by abnormalities in signal pathways associated with cell proliferation and tumor microenvironment. The current study provides evidence that the transcription factors including EZH2, EGR1, and SOX17 can be developed as the promising targets used for the molecular targeted therapy in patients with HCC.

## Data Availability Statement

Publicly available datasets were analyzed in this study. This data can be found here: [https://www.ncbinlm.nih.gov/geo/ (GSE76427, GSE36376, GSE84005, and GSE101685)], [https://jhubiostatistics.shinyapps.io/recount/ (“TCGA_liver” and “gtex_liver”)], and [http://lifeome.net/database/hccdb/ (ICGC LIRI-JP)].

## Author Contributions

XG performed the data extraction, statistical analysis, and drafted the manuscript. YL, GD, JL, and XL assisted in literature investigation and data validation. XZ and AL supervised the literature investigation, statistical analysis, and reviewed the manuscript. All authors have read and agreed to the published version of the manuscript.

## Conflict of Interest

The authors declare that the research was conducted in the absence of any commercial or financial relationships that could be construed as a potential conflict of interest.

## Footnotes

1 https://www.ncbi.nlm.nih.gov/geo/

2 https://www.gtexportal.org/home/

3 https://jhubiostatistics.shinyapps.io/recount/

4 http://www.broad.mit.edu/gsea

5 http://www.R-project.org

## Abbreviations

AUC: area under the curve
CI: confidence intervals
DEGs: differentially expressed genes
DP.eHCC: diagnosis prediction model for early HCC
GeNets: genome networks
GSEA: Gene Set Enrichment Analysis
HCC: hepatocellular carcinoma
HR: hazard ratio
ML: machine learning
SP.eHCC: survival risk prediction model for early HCC
WGCNA: weighted correlation network analysis.
