# Supplementary material for: Identification of Gene Signatures for Diagnosis and Prognosis of Hepatocellular Carcinomas Patients at Early Stage
Source: Front Genet. 2020 Jul 30;11:857. doi: 10.3389/fgene.2020.00857 (PMC7406719; doi:10.3389/fgene.2020.00857)
Supplement: Supplementary file 1 [file Presentation_1.pdf]

Supplementary Material

1 Supplementary Data

Supplementary Material should be uploaded separately on submission. Please include any supplementary data, figures and/or tables. All supplementary files are deposited to FigShare for permanent storage and receive a DOI.

Supplementary material is not typeset so please ensure that all information is clearly presented, the appropriate caption is included in the file and not in the manuscript, and that the style conforms to the rest of the article. To avoid discrepancies between the published article and the supplementary material, please do not add the title, author list, affiliations or correspondence in the supplementary files.

2 Supplementary Figures and Tables

For more information on Supplementary Material and for details on the different file types accepted, please see here. Figures, tables, and images will be published under a Creative Commons CC-BY licence and permission must be obtained for use of copyrighted material from other sources (including re-published/adapted/modified/partial figures and images from the internet). It is the responsibility of the authors to acquire the licenses, to follow any citation instructions requested by third-party rights holders, and cover any supplementary charges.

2.1 Supplementary Figures

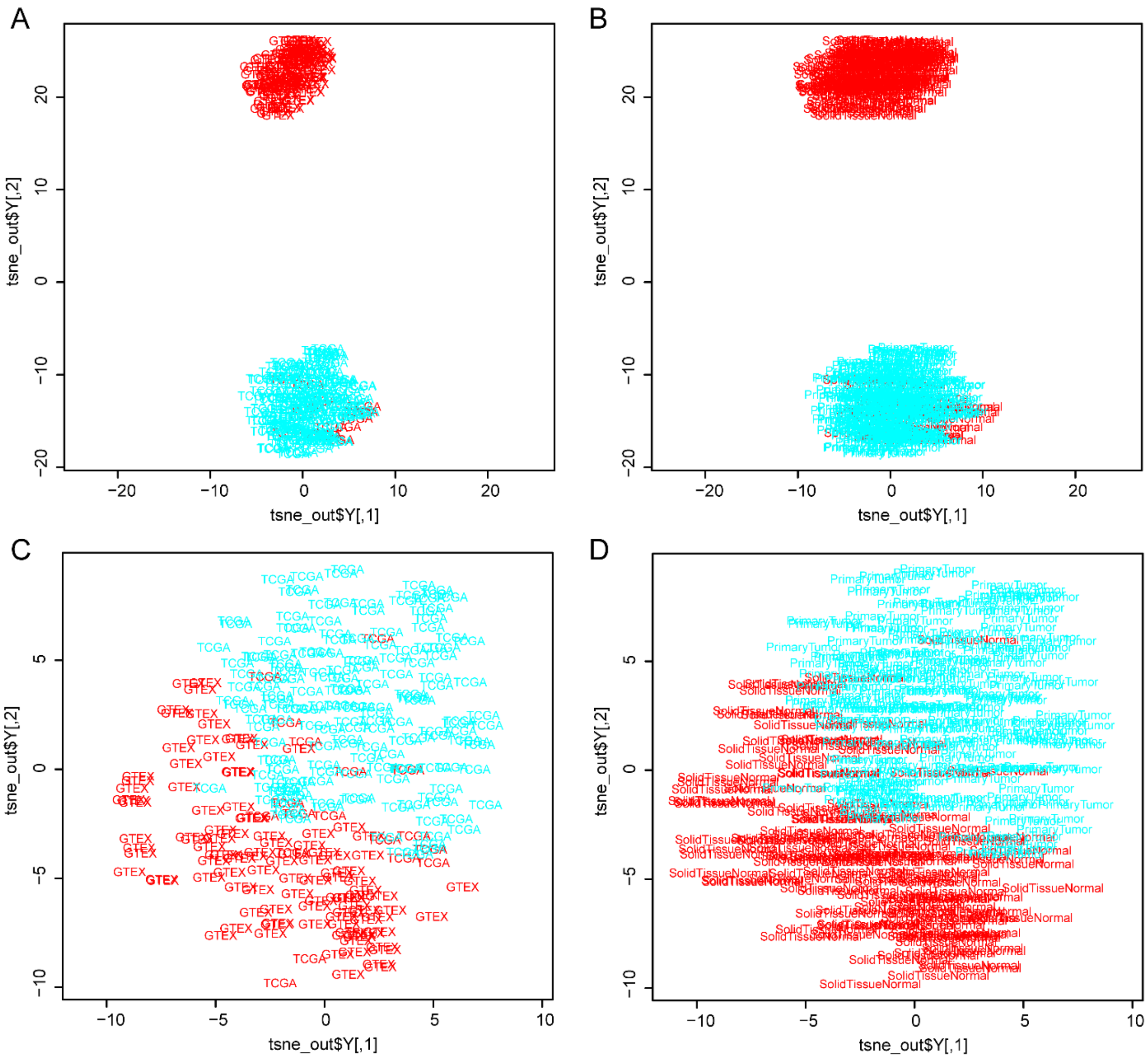

Supplementary Figure 1. Visualization and adjustment of batch effects between TCGA and GTEx. (A) tSNE analysis of merged transcription data of TCGA and GTEx defined by the data source (before batch effect corrected). (B) tSNE analysis of merged transcription data of TCGA and GTEx defined by the tissue type (before batch effect corrected). (C) tSNE analysis of merged transcription data of TCGA and GTEx defined by the data source (after batch effect corrected). (D) tSNE analysis of merged transcription data of TCGA and GTEx defined by the tissue type (after batch effect corrected).

A

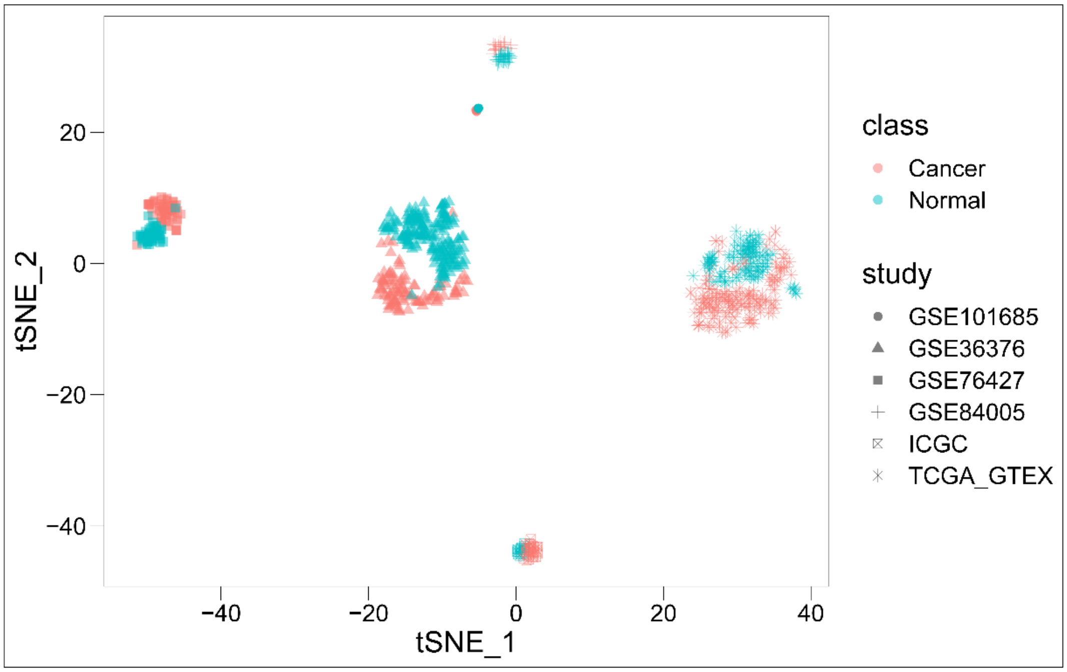

B

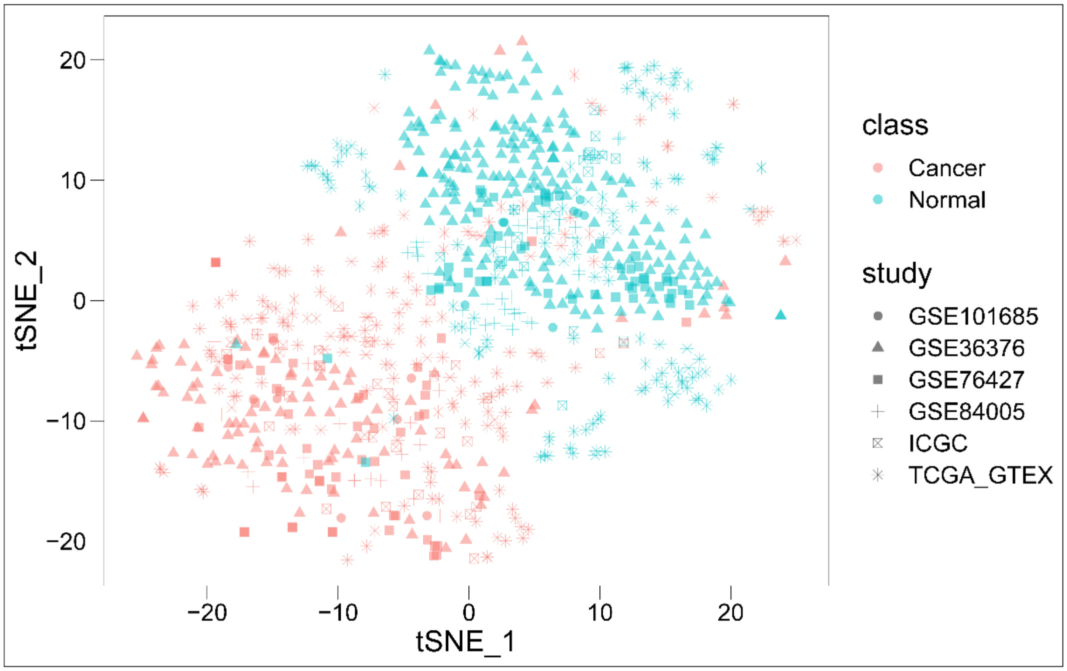

Supplementary Figure 2. Visualization and adjustment of batch effects among 6 eligible datasets. (A) tSNE analysis of merged transcription data of 6 eligible datasets labeled as the classification of tissue and study (before batch effect corrected). (B) tSNE analysis of merged transcription data of 6 eligible datasets labeled as the classification of tissue and study (after batch effect corrected).

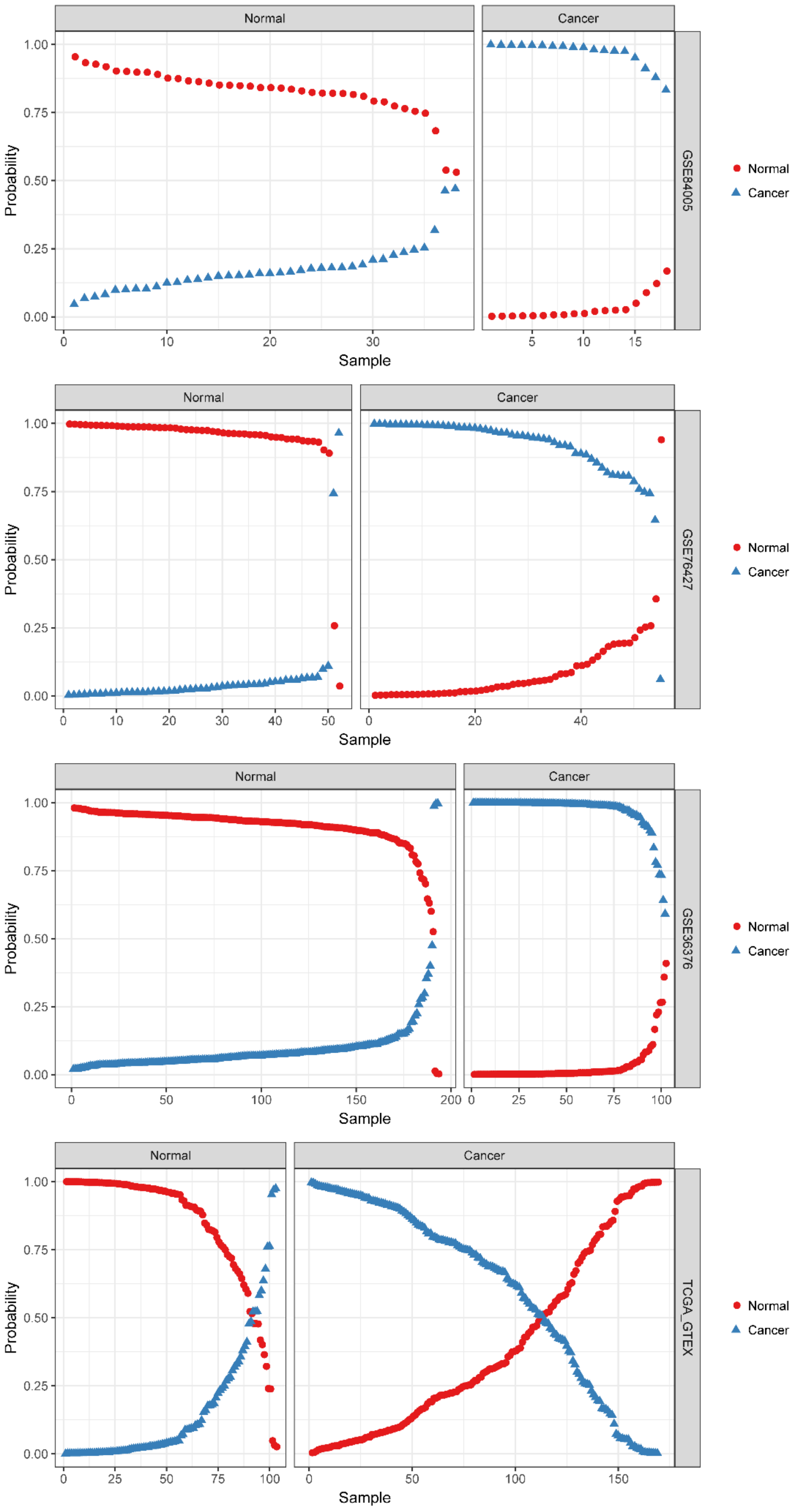
Normal
Cancer
Probability
1.00
0.75
0.50
0.25
0.00
Sample
GSE84005
GSE76427
GSE36376
TCGA_GTEX
Normal
Cancer

Supplementary Figure 3. Cross-validation of the binomial classifier on training datasets. Estimated probabilities for samples in testing datasets (GSE101685 and ICGC). For each sample, there are two points, corresponding to the probability that the sample belongs to the respective class. Within each dataset and class, samples are sorted by the probability of the true class. For most samples, the probability of the true subtype is near 1, indicating an unambiguous classification.

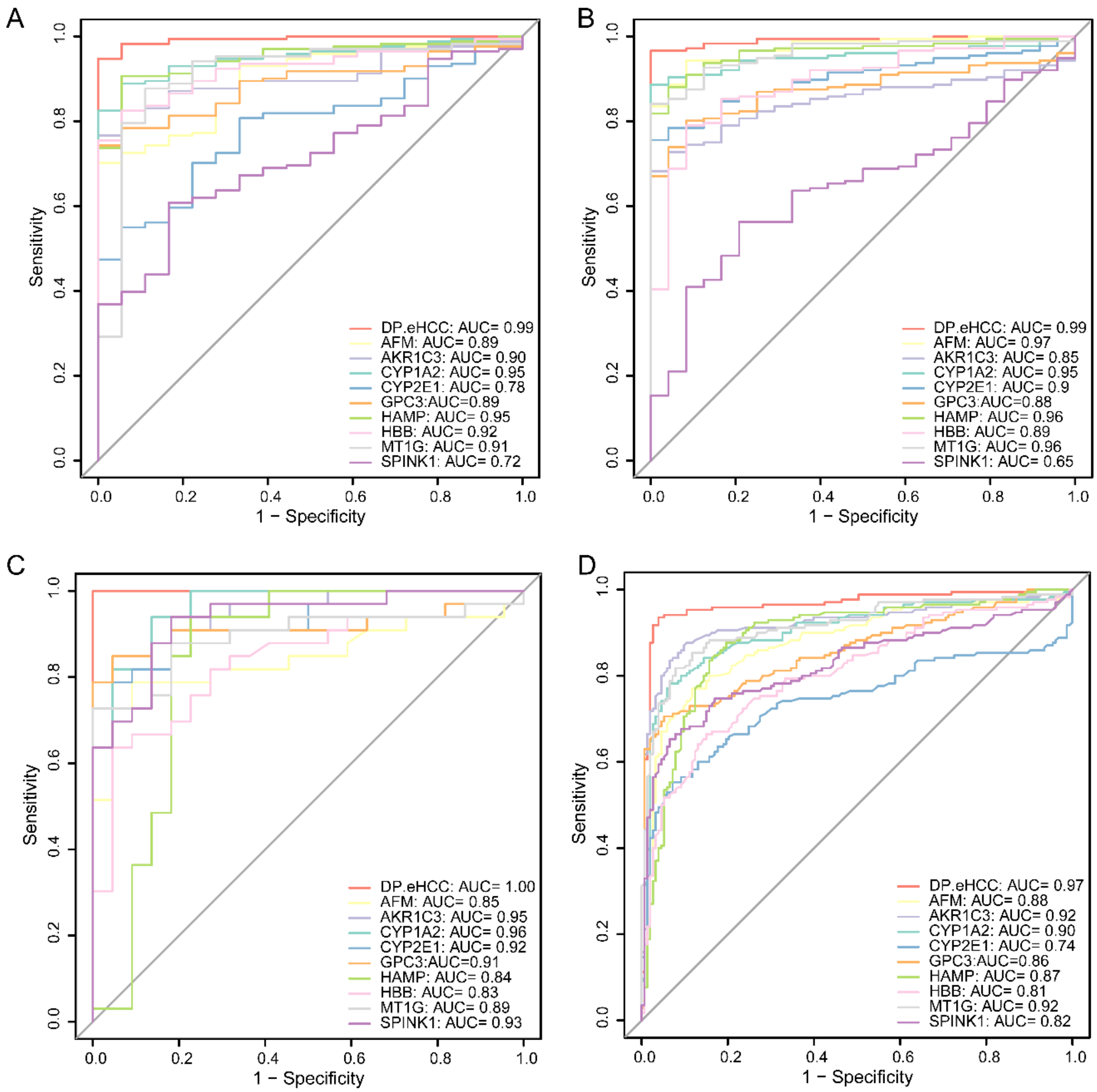

Supplementary Figure 4. Validation of the diagnosis performance of the DP.eHCC model and its gene members in different stages of HCC from TCGA and ICGC database. (A) Receiver operating characteristic (ROC) curve analyses of DP.eHCC model (AUC = 0.9935, $p < 0.001$) and its gene members in 189 early HCC of TCGA. (B) Receiver operating characteristic (ROC) curve analyses of DP.eHCC model (AUC = 0.9915, $p < 0.001$) and its gene members in 200 advanced HCC of TCGA. (C) Receiver operating characteristic (ROC) curve analyses of DP.eHCC model (AUC = 1.00, $p < 0.001$) and its gene members in 55 early HCC of ICGC. (D) Receiver operating characteristic (ROC) curve analyses of DP.eHCC model (AUC = 0.9653, $p < 0.001$) and its gene members in 323 advanced HCC of ICGC.

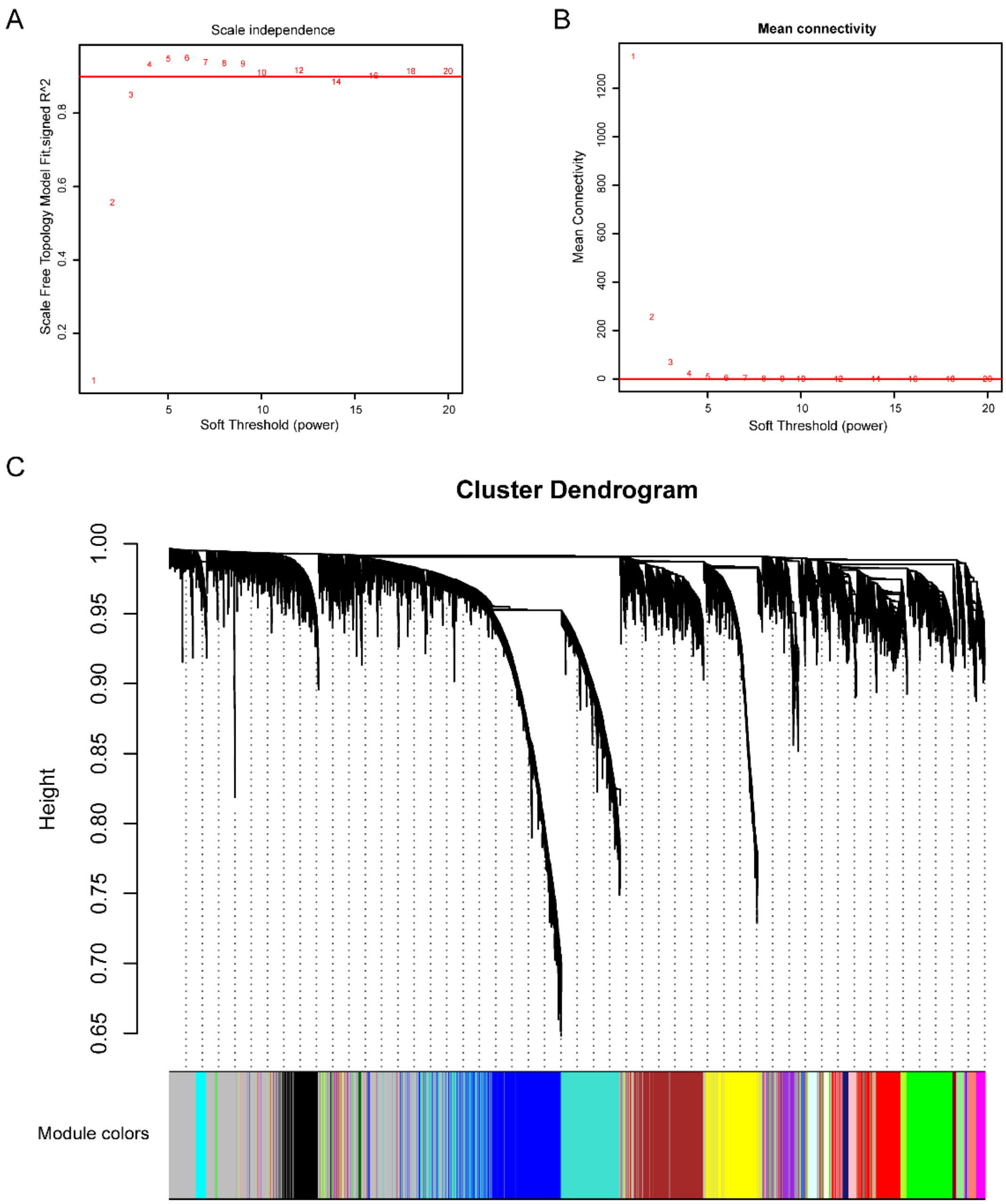

Supplementary Figure 5. Building a co-expression network by WGCNA analysis based on the transcriptome profile of mRNA in early HCC. (A-B) Soft-thresholding power of 3 was automatically generated in the scale free topology model. (C) A total of eventually 22 co-expression modules were identified in dendrogram of hierarchical clustering.

2.2 Supplementary tables

Supplementary Table 1. Main features of 826 early HCC patients from eligible datasets included in the machine learning and bioinformatics analyses.

Supplementary Table 2. The coefficients of gene signatures in the classifier analyzed by elastic net for each class.

Supplementary Table 3. Data for cross-validation of the binomial classifier on training datasets.

Supplementary Table 4. Data for cross-validation of the binomial classifier on testing datasets.

Supplementary Table 5. Prognosis-related genes screened by univariate Cox regression.
